# High-performance liquid chromatography separation and intact mass analysis of detergent-solubilized integral membrane proteins

**DOI:** 10.1016/j.ab.2010.11.008

**Published:** 2011-03-15

**Authors:** Georgina Berridge, Rod Chalk, Nazzareno D’Avanzo, Liang Dong, Declan Doyle, Jung-In Kim, Xiaobing Xia, Nicola Burgess-Brown, Antonio deRiso, Elisabeth Paula Carpenter, Opher Gileadi

**Affiliations:** Structural Genomics Consortium, University of Oxford, ORCRB, Roosevelt Drive, Oxford OX3 7DQ, UK

**Keywords:** Integral membrane protein, HPLC, Electrospray ionization mass spectrometry, Methanol, Detergent, Intact mass analysis, LC–MS, Isocratic elution

## Abstract

We have developed a method for intact mass analysis of detergent-solubilized and purified integral membrane proteins using liquid chromatography–mass spectrometry (LC–MS) with methanol as the organic mobile phase. Membrane proteins and detergents are separated chromatographically during the isocratic stage of the gradient profile from a 150-mm C3 reversed-phase column. The mass accuracy is comparable to standard methods employed for soluble proteins; the sensitivity is 10-fold lower, requiring 0.2–5 μg of protein. The method is also compatible with our standard LC–MS method used for intact mass analysis of soluble proteins and may therefore be applied on a multiuser instrument or in a high-throughput environment.

Integral membrane proteins represent approximately 25% of the human proteome and are of crucial biological importance in regulating the composition of the cell and the extracellular environment, membrane potential, metabolism, cell structure, and signaling pathways. As a result of their position as the gateway to cells, they are also the targets for many drugs, in fact more than 50% of commercially available small molecule drugs target membrane proteins such as G-protein-coupled receptors, ion channels, transporters, other receptors, and enzymes [1]. Despite the importance of membrane proteins in biology and pharmacology, to date only 246 crystal structures exist for membrane proteins [2] in comparison to over 50,000 for soluble proteins, and only 14 of those structures are of human integral membrane proteins. The difficulties in expression, extraction, and isolation of homogeneous membrane proteins of high quality and quantity for crystallization trials have been well documented [3,4]. Analysis of the proteins produced is also difficult. In particular, SDS–PAGE^5^ provides very poor estimates of the molecular mass of membrane proteins.

Intact mass analysis by MS has proved to be an invaluable method for routine protein identification and quality assessment in the high-throughput production of soluble proteins for crystallography [5,6]. Published LC–MS techniques exist for intact mass analysis of integral membrane proteins using nonstandard mobile and stationary phases (for example, Refs. [7–10]). These methods are not easily adapted to routine mass spectrometric analyses and have not been widely adopted. We have developed a simple and robust LC–MS method for routinely determining accurate mass to within 1.0 Da of the calculated mass for integral membrane proteins, with a sensitivity ∼10-fold lower than our existing methods for soluble proteins. This fast and convenient method does not require pretreatment or protein precipitation for removal of detergents. Moreover, the method may be used interchangeably with soluble protein analysis on the same LC–MS instrument.

## Materials and methods

### Reagents

LC–MS grade reagents were purchased from Fluka (Sigma–Aldrich, UK). Detergents were purchased from Anatrace (Maumee, OH, USA). All other reagents were analytical grade purchased from Sigma–Aldrich.

### Cloning and expression of integral membrane proteins

Proteins from a variety of membrane protein families, including channels, transporters, and enzymes, were expressed in bacterial, yeast, or baculovirus-infected insect cell cultures using a variety of expression vectors and cell lines. All proteins examined were expressed as fusions to oligohistidine or FLAG tags. The proteins were extracted from whole cells or from membrane preparations using detergents, and were purified by immobilized metal affinity chromatography (IMAC) or M2 anti-FLAG agarose affinity purification as appropriate. The protein was further separated from contaminants using gel filtration chromatography, using a gel filtration buffer (typically 10–20 mM HEPES or Tris, pH 7.5, 150 mM NaCl or KCl, 0–10% glycerol, ±DTT) in the presence of appropriate detergent and in one instance (SCD; stearoyl-CoA desaturase) the addition of a known lipid mix. Where noted, the purification tag was cleaved using TEV protease. In some cases, the purified proteins were finally concentrated using either 50- or 100-kDa molecular weight cutoff Centricon (Millipore, Watford, UK) concentrator to between 0.1 and 6 mg/ml.

### Chromatography

Reversed-phase chromatography was performed in-line prior to mass spectrometry using an Agilent 1100 HPLC system. Protein was diluted (between 5-fold and 20-fold in different experiments) with a solution of 1% formic acid to an injection volume of 10 μl and loaded on to a 4.6 mm internal diameter Zorbax 300SB-C3 column with column length of 50, 150, or 200 mm. Effective column length was extended by serial attachment of 50- and 150-mm columns. The solvent system used consisted of 0.1% formic acid in ultrahigh purity water (Millipore) (solvent A) and 0.1% formic acid in methanol (solvent B). The details of chromatography varied in different experiments as indicated in parentheses. Proteins were injected at initial conditions of 95% A and 5% B and a flow rate of 0.5 ml/min. After 1 min at 5% B, an initial linear gradient from 5% B to 95% B was applied, for 7 min (2–13 min). Elution then proceeded isocratically at 95% B for 10 min (3–15 min) before applying a second linear gradient from 95% B to 5% B over 2 min. Equilibration at 5% B for 2–4 min returned the system to the initial conditions (the times were varied in different experiments, as indicated in the figures). Chromatography was performed in a column oven set to 40 °C. The total amount of protein loaded varied from 0.2 to 6.0 μg.

### Mass spectrometry

Protein intact mass was determined using an MSD-ToF electrospray ionization orthogonal time-of-flight mass spectrometer (Agilent). The instrument was configured with the standard ESI source and operated in positive-ion mode. The ion source was operated with the capillary voltage at 4000 V, nebulizer pressure at 50 psig, drying gas at 350 °C, and drying gas flow rate at 10 L/min. The instrument ion optic voltages were as follows: fragmentor 250 V, skimmer 60 V, and octopole RF 250 V. These parameters are identical to our standard methods for intact mass measurement of soluble proteins.

### Data analysis

Data analysis was performed using the MassHunter Qualitative Analysis Version B.01.03 Build 1.3.157.0 (Agilent) software. Individual LC scans were inspected and selected manually based on signal to noise and on the presence of a characteristic protein ionization series with at least five charge states. Varying numbers of scans were combined depending on the width of the chromatographic peak. Deconvolution was performed between 200 *m*/*z* and 3500 *m*/*z*, using peaks with a ratio of signal to noise greater than 30:1. The mass range for deconvolution was 10,000–100,000 Da and the step mass was 1 Da. Average mass was calculated at 90% of the peak height using a minimum of five consecutive charge states and a minimum “protein fit” score of 8.

The technique used was to “walk” through the total ion chromatogram (TIC) one scan at a time, searching each individual *m*/*z* spectrum for a characteristic protein-like multiple ionization envelope. The initial walk through was used to determine the chromatographic characteristics of the detergent and to eliminate those regions where detergent is presumed to have caused detector saturation. Those spectra found to include a protein ionization envelope were combined and deconvoluted. Typically membrane proteins elute directly prior to or directly after the detergent, and are often coelute with some associated detergent. The ionization of the detergent species may then mask the protein ionization. When this was noted, axis zoom was used to clearly visualize the protein ionization spectrum, and only an area between the detergent species was used for deconvolution. Deconvolution of the *m*/*z* spectrum to neutral charge state was achieved using the set parameters described. The deconvoluted mass was compared with the theoretical mass derived from the DNA sequence of the expression construct for each protein. Where significant discrepancies between observed and theoretical mass occurred, these differences in mass were compared with expected mass changes for known modifications in the Unimod database (http://www.unimod.org).

### Tryptic digestion and phosphorylation mapping

The identity of proteins in solution or in gel bands excised following SDS–PAGE was confirmed by tryptic digestion and tandem MS (LC–MS/MS). SDS–PAGE gel bands were excised as 1 × 4-mm slices using a gel cutting tip (GeneCatcher, Web Scientific) and stored in 10% MeOH at 4 °C. Prior to digestion, the methanol solution was removed and replaced with 100% acetonitrile for 2–5 min. The solution was then removed and replaced with 100 μl of 100 mM NH_4_HCO_3_ (pH 8.0). For digestion of proteins in solution, an aliquot (<30 μl) of the protein was added directly to 100 μl of 100 mM NH_4_HCO_3_. For phosphopeptide analysis, 30 μl of protein (0.5 mg/ml) in solution was diluted to 100 μl with 100 mM NH_4_HCO_3_ (pH 8.0). In all cases, 1 μl of 1 M dithiothreitol was added and incubated at 56 °C for 40 min. Four microliters of 1 M iodoacetamide was then added and the reaction incubated at ambient temperature in the dark for 20 min. A further 1 μl of 1 M dithiothrietol, 200 μl of 100 mM NH_4_HCO_3_, and 1 μl of trypsin solution (sequencing grade, Sigma Cat. No. T 6567, 1 mg/ml in 0.01 M HCl) was then added. Tryptic digestion proceeded at 37 °C for 16 h and was terminated by the addition of 3 μl of formic acid. LC–MS/MS was performed using a Dionex U3000 nano HPLC coupled to a Bruker Esquire HCT ion trap mass spectrometer. The amount of 1–5 μl of tryptic digest was loaded on to a 200 μm i.d. × 5 cm PS-DVB monolith column (Pepswift, Dionex Corp.) A linear gradient of 0% B to 15% B was developed over 5 min, followed by a second linear gradient from 15% B to 40% B over 2 min. The column was washed at 90% for 2 min and then equilibrated at 0% B for a further 6 min. Solvent A was 2% (v/v) acetonitrile, 0.1% formic acid in water, solvent B was 80% acetonitrile, 0.1% formic acid. The flow rate was 2.5 μl/min. The mass spectrometer was operated in positive ion, standard enhanced mode with a scan rate of 8100 *m*/*z*/s and a scan range of 250–1800 *m*/*z*. The trap accumulation time was 200 ms and the accumulation target was 200,000 counts. Data-dependent peptide fragmentation was performed in Auto MSMS mode.

Phosphorylation mapping of protein KCNJ12 was performed as follows: phosphopeptide enrichment and tandem mass spectrometry were performed using the same instrumentation as for general peptide MS/MS. A total of 75 μl of KCNJ12 tryptic digest was loaded sequentially on to a 300 μm i.d. × 2-mm TiO_2_ precolumn (5-μm particle size, Titanshphere, GL-Sciences, Japan) followed by a linear gradient of 0% B, 10% C to 90% B, 10% C over 14 min to elute nonphosphorylated peptides. Solvent A was 2% (v/v) acetonitrile, 0.1% formic acid in water, solvent B was 80% acetonitrile, 0.1% formic acid, and solvent C was 100% formic acid, with a flow rate of 3 μl/min. Phosphopeptides were eluted from the precolumn by injection of 5 μl of ammonium hydroxide solution (28% NH_3_) from a sealed vial, and eluted isocratically in 100% solvent (A). Eluted phosphopeptides were passed directly to the electrospray source without further chromatography. Mass spectrometer parameters were as described above. Compound extraction and peptide deconvolution were performed using the DA data analysis program (Bruker Daltonik). Database searching was performed using the Mascot 2.2.04 search algorithm (Matrix Science) with the following search parameters: charge states +2, +3; MS tolerance 1.5 Da; MSMS tolerance 0.5 Da; UniProt/SwissProt database without taxonomic restrictions.

## Results

### Effect of methanol as mobile phase B

**Fig.1:**
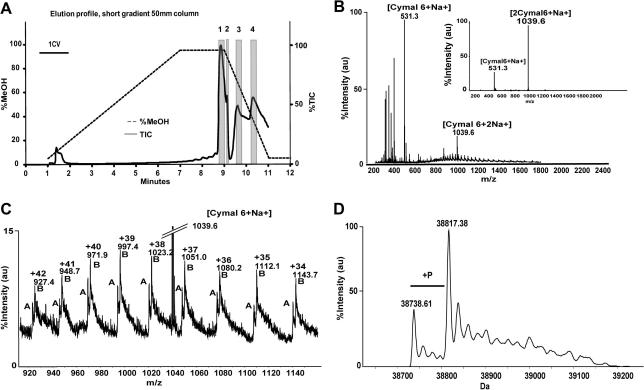
Chromatogram and mass spectra of purified KCNJ12. (A) Total ion current (TIC) elution profile for KCNJ12 purified in the detergent Cymal-6, eluted with methanol. Dotted line: methanol concentration. Solid line: TIC (arbitrary units). The gray rectangles denote regions of the chromatogram (regions 1–4) referred to in the text. (B) *m*/*z* spectrum of region 4; combination of detergent signals (Cymal 6) and a protein-like spectrum. Inset: *m*/*z* spectrum of regions 2 + 3, identified as Cymal-6. (C) *m*/*z* spectrum of region 4, enlarged segment of the spectrum shown in (B), showing a typical protein ion series. (D) Mass spectrum of KCNJ12 after deconvolution.

Initial attempts to obtain intact mass for membrane proteins by LC–MS using our standard protocols for soluble proteins were unsuccessful. We found, however, that by replacing the organic acetonitrile phase with methanol we could record an accurate intact mass for the potassium channel KCNJ12 [11] (Fig. 1). A complex total ion current pattern appeared late in the chromatogram; a careful analysis of individual spectra was used to resolve specific features within this region of the chromatogram. The first major component appeared to be TEV protease (chromatogram region 1 in Fig. 1A, elution time between 8.7 and 9.0 min); the presence of TEV protease was verified by deconvolution (data not shown). There followed a short peak of the detergent Cymal-6 (the major component of regions 2 and 3, elution time between 9.106 and 9.160 min), and then a dip in the TIC which appears to be detector saturation between 9.232 and 9.341. Between 10.409 and 10.698 min (region 4) another protein envelope was observed (Fig. 1B and C). The deconvoluted mass spectrum shows one peak at 38738.6 Da, which corresponds to the accurate mass of the KCNJ12 protein after TEV cleavage (Fig. 1D). However, the most prominent peak indicates a mass of 38817.4 Da. The mass difference of +78.8 Da suggests a phosphorylation. This was later confirmed by tryptic digest and phosphopeptide analysis, mapping the phosphorylation site to either Thr-353 or Ser-354 (data not shown). Phosphorylation of KCNJ12 at Thr-353 was suggested previously [12] based on site-directed mutagenesis.

These results indicated that accurate mass analysis of membrane proteins was feasible. The key seemed to be the use of methanol as the mobile phase, and the careful search of multiple individual spectra for a characteristic protein ionization envelope. However, the analyses were not easy to reproduce with KCNJ12 or with other proteins (the initial success may have been associated with some unusual properties of a particular protein batch, e.g., purity or detergent:protein ratio). Furthermore, the elution of protein peaks after the isocratic segment of the chromatogram indicated that there was scope for optimization of the chromatographic procedure.

### Effect of extended methanol gradient elution

Examination of the total ion chromatograms from the analyses of KCNJ12 (Fig. 1) and a second membrane protein HVCN1 (data not shown) clearly showed that elution of both proteins and detergent occurs toward the end or after the 3 min 95% methanol isocratic section. We attempted three modifications of the chromatographic protocol: shortening the initial gradient, extending the length of the isocratic section at 95% B, and using longer columns.

**Fig.2:**
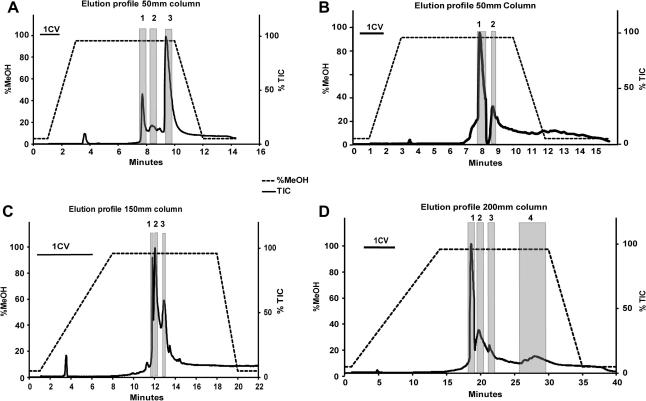
Optimization of gradient and column lengths. (A) KCNJ12 elution profile: 50-mm column, 12 min gradient, detergent, Cymal-6. (B) SCD elution profile: 50-mm column, 16 min gradient/isocratic, detergent, DDM. (C) SCD elution profile: 150-mm column 22 min gradient/isocratic, detergent, DDM. (D) SCD elution profile: 200-mm column, 40 min gradient/isocratic, detergent, DM + lipids.

The elution protocol was modified by shortening the initial gradient to 2 min (1.2 CV) rather than 6 min and extending the 95% methanol isocratic section to 7 min (4.2 CV). This method was applied to KCNJ12 (Fig. 2A) and SCD (Fig. 2B). This new LC–MS method was shown to be more reliable and able to separate membrane proteins from detergent. This separation occurred entirely over the isocratic section. Elution of salts was observed during the initial gradient phase. Step elution in 95% methanol plus 0.1% formic acid was attempted but proved unsuccessful (data not shown) as the detergent and protein eluted over a narrow section of the TIC and detector saturation was still observed.

### Effect of column length

As another route to improve the chromatographic separation of membrane proteins and detergents we tested longer C-3 columns (150 and 200 mm versus 50 mm). The initial methanol gradient was developed over 7 min (1.4 CV and 1.05 CV for the 150- and 200-mm columns, respectively) and the 95% methanol isocratic section was extended to 10 min (2 CV and 1.5 CV, respectively). Overall, this improved the chromatographic separation as shown for SCD (Fig. 2C and D). Two other proteins, HVCN1 and ZMPSTE24, also showed improved separation on the longer columns (data not shown). The different species of detergent were also separable using the longer column and the extended isocratic stage (Table 1). Further extension to 200 mm also proved beneficial and increased the separation of the free detergent from the protein without detector saturation. Furthermore, we observed the separation of excess free lipids from protein isolated in the presence of detergent and lipid species (Fig. 4).

The details of the chromatograms vary between samples. Although we generally find that free detergents elute early, followed by proteins, and then other contaminants, the details of elution times, peak shape, and size vary between different samples. It is crucial, therefore, to carefully scan the *m*/*z* spectra throughout the chromatogram to identify the region containing data on protein *m*/*z*.

### Mass accuracy and sensitivity

Using methanol gradient elution and one of the three chromatographic protocols outlined above we obtained LC–MS data and intact mass values for seven integral membrane proteins from five distinct families, purified in the presence of six different detergents. The choice of detergent was dependent on the protein purification, rather than as a parameter for LC–MS. The observed masses compared with theoretical mass for each protein, using data from 28 separate measurements, are shown in Table 2. In all cases where the mass difference (Δ*M*) was ⩾2 Da, a plausible interpretation could be provided: most often, the removal of the initiator methionine [13], with an occasional acetylation or carboxylation. These enzymatic posttranslational modifications are commonly observed for the expression systems used (Refs. [14–16] and unpublished observations). Taking these presumed posttranslational modifications into account, the mean mass accuracy for proteins ranging in size from 34 to 67 kDa was ±2 Da and comparable to that observed for soluble proteins using standard methods. Since the predicted mass and identity of each protein was known in advance, in all cases the mass accuracy was deemed sufficient to confirm the identity and integrity of the analyzed protein, as well as providing a tentative identification of modification states.

### Detergents and protein–detergent association

**Fig.3:**
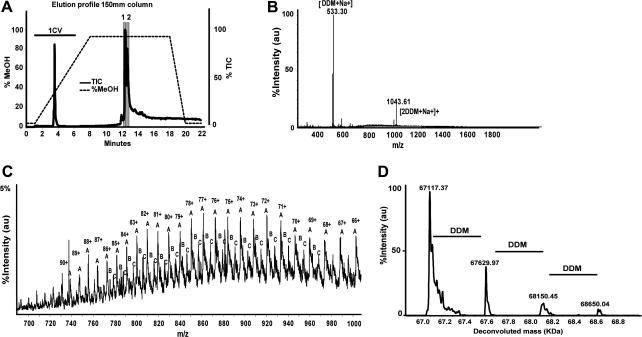
Detection of stripped protein and detergent adducts. (A) TIC elution profile of ABCB10. (B) *m*/*z* spectrum of region 2 of the chromatogram. The free detergent ions dominate the spectrum, dwarfing the protein ionization signal. (C) *m*/*z* spectrum of region 2, magnified segment of figure (B). (D) Deconvoluted mass spectrum of region 2 of the chromatogram, showing the accurate intact mass, as well as adducts with 1, 2, and 3 detergent molecules.

**Fig.4:**
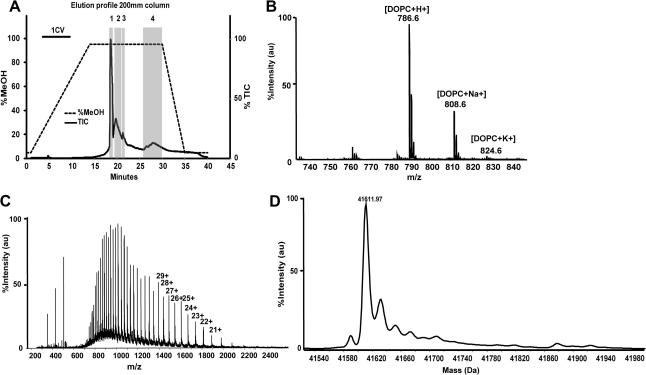
Detection of protein and lipids. (A) TIC elution profile of SCD purified in the presence of a lipid/detergent mix. (B) Chromatogram region 4 of the chromatogram: ionization of lipid species (regions 2 and 3 contain detergents and contaminants; not shown). (C) *m*/*z* spectrum of region 1, illustrating the separation of the protein from the lipids and detergents. Note that protein spectra are rarely as clean as this; compare Fig. 3B. (D) Deconvoluted mass spectrum.

In addition to accurate mass spectra for integral membrane proteins, it was possible to obtain spectra for free detergent species in the solution and to identify detergent species which appear to be noncovalently associated with the membrane protein. Fig. 3 shows an example of protein-associated detergent molecules. The deconvoluted mass spectrum of the ATP binding cassette protein ABCB10 shows a mass of 67117.37 Da, which we interpret as the predicted mass after methionine removal and acetylation. Three additional peaks match the protein mass plus 1, 2, or 3 molecules of dodecyl maltoside, the detergent used in purification. In addition to protein-associated detergents, free detergents and lipids are clearly seen in *m*/*z* spectra: for example, the peaks of DDM in Fig. 3B, of Cymal-6 in Fig. 1B, and of lipids (DOPC, dioleoylphosphatidylcholine) in Fig. 4B. Note that the detergent peaks often dominate the *m*/*z* spectrum (as in Fig. 3B), making the protein spectrum hard to detect in a casual inspection. Table 1 lists the ions we observed in spectra of detergent-containing samples. These data can be useful in monitoring the efficiency of detergent exchange; it is very important to verify that the LC column is completely washed of detergent residues, as these tend to persist and appear in subsequent samples.

### Limitations and practical considerations

The largest protein for which we obtained mass spectra was of 67 kDa; attempts to analyze significantly larger proteins (140–250 kDa) failed. This is commonly observed with soluble proteins, although the size limitation is unlikely to be absolute. Similarly, we have obtained experimental masses of proteins purified in some detergents (DM, DDM, Cymal-6, FC14, FC16, OG ± cholesterol hemisuccinate), but not with other detergents, including LDAO and C18E8.

The concentrations of the protein and the detergent are also important for success. Protein samples as low as 0.27 μg could be analyzed, although more typically 1–5 μg was required (protein concentrations above 0.2 mg/ml have been successful). Many detergents are concentrated along with the protein in standard centrifugal concentrators, even when 100-kDa cutoff membranes are used. The high detergent concentrations, often >0.5%, suppress the protein signal. One possible way to alleviate this problem is to purify the protein by gel filtration in a buffer containing no more than three times the critical micelle concentration of the protein, then take a sample of the highest concentration fraction from the gel filtration for LC–MS analysis, avoiding the accumulation of detergents in a spin concentrator.

**Fig.5:**
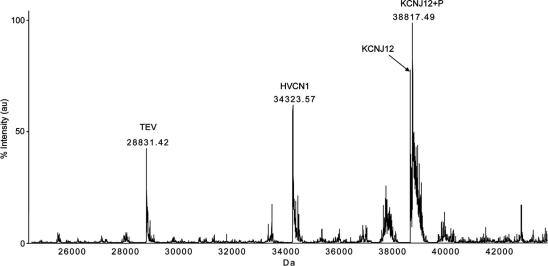
Intact mass analysis of a mixed population of proteins. A mixture of the membrane protein KCNJ12, the soluble TEV protease used to cleave the fusion tag, and the membrane protein HVCN1, which occurred as a contaminant from an earlier analysis on the same LC column.

Finally, the purity of the protein is an important factor; because of the complexity of the protein–detergent spectra it is difficult to deconvolute mixed or heterogeneously degraded proteins. However, in cases where the spectra are exceptionally clean, we have been able to resolve separate proteins in a mixed ion population (Fig. 5).

### Protein confirmation by tryptic peptide analysis

As an alternative method of protein identification we have applied tryptic digest followed by tandem MS on an ion trap. The procedures were the same as those used for soluble proteins. This analysis could be applied to purified proteins as well as isolated gel bands from impure preparations. A summary of MS/MS data obtained for the proteins described in this work is shown in Table 3. The peptide coverage is low, typically limited to the hydrophilic regions of an integral membrane protein. MS/MS results are often sufficient for unequivocal identification of the protein, and are particularly useful in identification of gel bands from intermediate stages of purification. However, peptide data very rarely confirm the integrity of the protein and its posttranslational modification status. Intact mass and tryptic digest MS/MS are best viewed as complementary analytical techniques.

## Discussion

Accurate determination of the intact mass of proteins is an important tool in protein analysis. Whether purifying naturally expressed or recombinant proteins, knowledge of the intact mass can establish the identity of the protein as well as its integrity, and provide data on protein modifications. To achieve this, the analysis should be reproducible, of high mass accuracy, and (ideally) using standardized methods that allow routine application in a busy MS facility. Such capabilities have long existed for soluble proteins, and have been used as a major tool for identification and quality assurance in a high-throughput protein production [5,6].

There is a general perception that membrane proteins are incompatible with intact mass analysis by standard LC–MS. We have attempted to apply our standard LC–MS methods to integral membrane proteins, using acetonitrile as the mobile phase, but failed to produce protein mass spectra. There are several processes which may be contributing to the difficulties in membrane protein MS:

(i) Proteins may precipitate during the chromatography phase.
(ii) Proteins may bind tightly to the HPLC stationary phase and thus fail to elute.
(iii) Detergents may undergo preferential ionization and thereby suppress ionization of the associated membrane protein.
(iv) The proteins may remain associated with heterogeneous amounts of detergents.

Detergents are known to cause signal suppression and instrument contamination in LC–MS analysis and their use is often avoided. Previous reports have accomplished mass determination of membrane proteins by a number of approaches. Treatment of the samples prior to chromatography to remove detergents followed by direct infusion in formic acid or using a specialized LC setup enabled accurate mass analysis of bacteriorhodopsin and spinach thylakoid membrane proteins D1 and D2 [9,10,17]. Cadene and Chait [7] and Takayama et al. [8] demonstrated MALDI-MS analysis of integral membrane proteins with useful though rather lower mass accuracy. When the objective is to analyze multisubunit or protein–detergent complexes, direct infusion MS has been the method of choice [18–21]. In particular Barrera et al. [19] have shown that gas-phase ionization occurs for protein–detergent complexes in the native state, and that dissociation of these complexes can be initiated by manipulation of the collision cell voltages. The mass accuracy has been sufficient to determine the gas-phase interactions taking place, but unit mass accuracy, necessary for confirmation of sequence, has not been shown. The specially modified *m*/*z* range instruments used in this work appear to be limited to a resolution of only 3000 [22]. To our knowledge, neither LC-electrospray-ionization nor MALDI methods have been widely adopted. When faced with the challenge of integral membrane protein analysis most laboratories have chosen a “bottom-up” approach by LC–MSMS analysis of tryptic peptides (reviewed in Ref. [23]).

The initial observation of this study was prompted by a global shortage of acetonitrile, which led us to test the use of methanol as the organic phase for LC–MS of soluble proteins. Slightly longer retention times and peak broadening were observed for soluble proteins, but there was no loss in sensitivity or mass accuracy. In addition we found that there was considerably less carryover of proteins between sequential samples, which substantially improved our throughput for soluble proteins. We have therefore adopted methanol as the organic phase of choice for all our LC–MS analyses.

Application of methanol-based elution enabled us initially to measure the intact mass of the integral membrane protein KCNJ12. The difficulty of reproducing the initial result, and the appearance of the protein spectrum at the end of the programmed gradient indicated that the chromatographic method could be optimized. Because of practical considerations (the availability of limited quantities of a variety of proteins), we did not perform an exhaustive optimization for every protein, but the large number of analyses and the consistent results for a diverse set of membrane proteins indicate that the methods are robust and widely applicable. We can summarize the conclusions and resulting guidelines as follows:

(1) Chromatography using a short gradient from 5 to 95% methanol (in 0.1% formic acid), followed by a prolonged isocratic segment in 95% methanol/0.1% formic acid, provides effective separation of membrane proteins from detergents and other buffer components.
(2) Prolonging the isocratic segment, using longer columns, or a combination of both can improve the separation.
(3) To fully utilize the chromatographic separation, *m*/*z* spectra should be scanned individually to identify the segments with the best protein ionization signal. Even then, the protein spectrum may be dwarfed by the more abundant free detergent ions, so the spectra should be scrutinized carefully for weak signals. Results of experiments not discussed in detail indicate that optimization of the chromatographic separation has a greater impact than varying the MS parameters on our instrument.
(4) The errors in protein mass determination seem to be less than 2 Da, after taking into account plausible interpretations of larger mass differences. This accuracy allows us to make precise predictions as to the source of observed differences, which can then be tested by other means (for example, we have performed phosphopeptide mapping in one instance, but other methods such as N-terminal sequencing may be applied where appropriate).
(5) The ratio of protein to detergent and the amount of protein are important determinants of success; samples for analysis should be taken from steps in the purification protocol that generate high protein–detergent ratios. In particular, concentrating protein–detergent mixes using centrifugal ultrafiltration often leads to excessive levels of detergent. The nature of the protein is also important: in addition to an apparent size limitation (>67 kDa), there are marked differences in the quality and signal to noise of spectra of different proteins (e.g., Figs. 1B, 3B and 4C). It is not easy at this stage to define the precise characteristics of protein samples that correlate with successful mass spectra; such generalizations may become possible with the accumulation of a larger data set.
(6) The nature and homogeneity of the detergent are important. Maltosides and glucosides (such as DM, DDM), FC14, FC16, and Cymal-6 work well, whereas C12E8 C12E10, LDAO, Tween 20, and Triton X100 do not.
(7) The ionized proteins are almost completely stripped of bound detergent, although a variable fraction of ion complexes may contain 1–5 bound detergent molecules.
(8) Mass spectra from different regions of the chromatogram also provide valuable information on detergents and lipids, both free and protein-associated. Special care should be taken to avoid the effects of sample carryover, which are particularly pronounced with hydrophobic analytes.

In summary, we present a robust and widely applicable method for LC–MS analysis of the intact mass of integral membrane proteins. Unit mass accuracy allows identification of these proteins and their probable modifications. The method can be easily integrated in routine operation of an instrument used for both soluble and membrane proteins. Although we have not tested this, it is conceivable that the sensitivity may be improved by using narrow-bore or smaller columns. We expect that future extension of the method to analysis of more problematic proteins can be based on further optimization of the liquid chromatography step, including the use of alternative solvent systems or columns.

## Figures and Tables

**Table 1:** Abbreviated names and observed ionized species of detergents.

Detergent	Acronym	FW	Molecular formula	*M* exp. (Da)	M obs. (Da)	Interpretation	Δ*M* (Da)
*n-*Hexadecylphosphorylcholine	FC16	407.50	C_21_H_46_NO_4_P	408.3237	408.38	[FC16+H^+^]^+^	0.06
				815.6400	815.72	[2FC16+H^+^]^+^	0.08
				1244.9386	124.97	[3FC16+Na]^+^	0.03
*n-*Dodecyl-*β*-d-maltopyranoside	DDM	510.60	C_24_H_46_O_11_	533.2932	533.29	[DDM+Na]^+^	−0.01
				1043.5972	1043.59	[2DDM+Na]^+^	−0.01
*n-*Octyl-*β*-d-glucopyranoside	OG	292.40	C_14_H_28_O_6_	315.1778	315.19	[OG+Na]^+^	0.01
				607.3664	607.36	[2OG+Na]^+^	−0.01
*n-*Octyl-*β*-d-maltoside	OM	454.40	C_20_H_38_O_11_	477.2306	477.23	[OM+Na]^+^	0.00
				931.4720	931.48	[OM+Na]^+^	0.00
*n-*Decyl-*β*-d-maltopyranoside	DM	482.60	C_22_H_42_O_11_	505.2619	505.26	[DM+Na]^+^	0.00
				987.5646	987.54	[2DM+Na]^+^	−0.03
*n-*Dodecyl-*N*,*N*-dimethylamine-*N*-oxide	LDAO	229.41	C_14_H_31_NO	459.4884	459.72	[2LDAO+H]^+^	0.23
Octaethylene glycol monododecyl ether	C12E8	538.77	C_28_H_56_O_8_	538.4313	537.41	[C12E8]^+^	−1.03
*n-*Dodecylphosphocholine	FC12	351.50	C_17_H_38_NO_4_P	374.2431	373.26	[FC12+Na]^+^	−0.98
				725.4969	723.53	[2FC12+Na]^+^	−1.97
*n-*Tetradecylphosphocholine	FC14	379.50	C_19_H_42_NO_4_P	380.2924	380.38	[FC14+Na]^+^	0.09
				781.5595	781.59	[2FC14+Na]^+^	0.03
				1160.8447	1160.85	[3FC14+Na]^+^	0.01
6-Cyclohexyl-hexyl-*β*-d-maltoside	Cymal-6	508.50	C_24_H_44_O_11_	531.2776	531.28	[Cymal6+Na]^+^	0.00
				1039.5659	1039.58	[2Cymal6+Na]^+^	0.01

**Table 2:** Summary of MS results for seven proteins analyzed.

Protein	Family	Detergents	*M* pred. (Da)	*M* obs. (Da)	Delta *M* (Da)	Probable interpretation	Mass error	SD	*n*
KCNJ12	K-channel	Cymal 6, FC14	38738.1	38738.61	−0.51	Intact mass	−0.69	0.25	2
KCNJ12 (phosphorylated)	K-channel	Cymal 6, FC14	38738.1	38817.38	+78.8	+phosphate	−1.2		2
HVCN1	Proton channel	FC16	34454.1	34323.3	−130.8	−met	−0.38	0.29	9
SCD	Enzyme	DDM, DM	45386.1	45255.8	−130.33	−met	−0.66	1.80	6
ABCB10	ABC-transporter	DDM	67203.6	67117.3	−86.23	−met +COCH_3_	−1.65	1.5	2
ZMPSTE24	Enzyme	DDM	58102.4	57972.9	−129.5	−met	−1.58	0.68	2
SC4MOL	Enzyme	OG + CHS	36053.4	36097.0	43.6	+COOH	−0.1	0.68	2
SLC22A18	Organic cation transporter	DM	45048.5	45049.75	1.25	Intact mass	1.25	n/a	1

Each protein was provided in the detergents listed. *M* pred: average mass predicted from the sequence. *M* obs: result of MS analysis. Δ*M* is *M* obs – *M* pred. The mass error is the difference between *M* obs and the predicted mass of the modified protein. SD is the standard deviation of *n* independent determinations.

**Table 3:** Tryptic digest analysis of membrane proteins.

Protein	MOWSE score	Coverage (%)	Number of peptides
SCD	159	13	4
KCNJ12	375	33	19
HVCN1	336	30	7
ABCB10	1437	44	30
ZMPSTE24	670	33	18
CH25H	144	13	6
SLC22A18	189	15	6
SC4MOLA	NA	NA	NA

The results shown present the best data obtained for each protein.

## Footnotes

5 *Abbreviations used:* SDS–PAGE, sodium dodecyl sulfate–polyacrylamide gel electrophoresis; LC, liquid chromatography; MS/MS, tandem mass spectrometry; TIC, total ion current; TEV, tobacco etch virus; CV, column volume. Abbreviated detergent names are listed in Table 1; genes are identified by HUGO designations.
